# Supplementary material for: Dietary Mg Supplementation Decreases Oxidative Stress, Inflammation, and Vascular Dysfunction in an Experimental Model of Metabolic Syndrome with Renal Failure
Source: Antioxidants (Basel). 2023 Jan 27;12(2):283. doi: 10.3390/antiox12020283 (PMC9952257; doi:10.3390/antiox12020283)
Supplement: Supplementary file 1 [file antioxidants-12-00283-s001.zip › antioxidants-2158108-supplementary.pdf]

antioxidants

MDPI

Article

Dietary Mg supplementation decreases oxidative stress, inflammation, and vascular dysfunction in an experimental model of metabolic syndrome with renal failure

Rodrigo López-Baltanás[1,+], María Encarnación Rodríguez-Ortiz[1,2,+], Juan M. Díaz-Tocados[3], Julio M. Martinez-Moreno[1], Cristina Membrives[1], Cristian Rodelo-Haad[2,4], M Victoria Pendón Ruiz de Mier[2,4], Mariano Rodríguez[2,4], Antonio Canalejo[5,*], Yolanda Almadén[1,#], Juan Rafael Muñoz-Castañeda[2,4, #]

[1] Instituto Maimónides de Investigación Biomédica de Córdoba (IMIBIC), Reina Sofia University Hospital/University of Cordoba, Córdoba, Spain.
[2] Redes de Investigación Cooperativa Orientadas a Resultados en Salud (RICORS), Instituto de Salud Carlos III, Madrid, Spain.
[3] Biomedical Research Institute of Lleida (IRBLleida), Vascular and Renal Translational Research Group, Arnau de Vilanova University Hospital, Lleida, Spain
[4] Instituto Maimonides de Investigacion Biomédica de Córdoba (IMIBIC), Reina Sofia University Hospital/University of Cordoba, Unidad de Gestión Clinica Nefrología, Córdoba, Spain
[5] Department of Integrated Sciences/Research Center on Natural Resources, Health and Environment (RENSMA), University of Huelva. Spain
* Correspondence: Antonio Canalejo; e-mail: antonio.canalejo@dbasp.uhu.es;
\+ Rodrigo López-Baltanás and Maria Encarnación Rodríguez-Ortiz should be considered joint first author.
\# Yolanda Almadén and Juan R. Muñoz-Castañeda should be considered joint senior author.

1. **Supplementary Figure S1:** Pathway enrichment of identified proteins. A,C,E,F,H,J,L,N) show pathway enrichment of proteins identified in the different experimental groups. Pathway enrichment among differentially expressed proteins was conducted using pathway classifications from the Reactome resource. With the aim to search for proteins networking STRING protein network analysis was performed with the 22 proteins differentially expressed between groups. (B,D,G,I,K,M,O) show interaction of proteins in each studied pathway. Protein network analysis was performed by using STRING software.

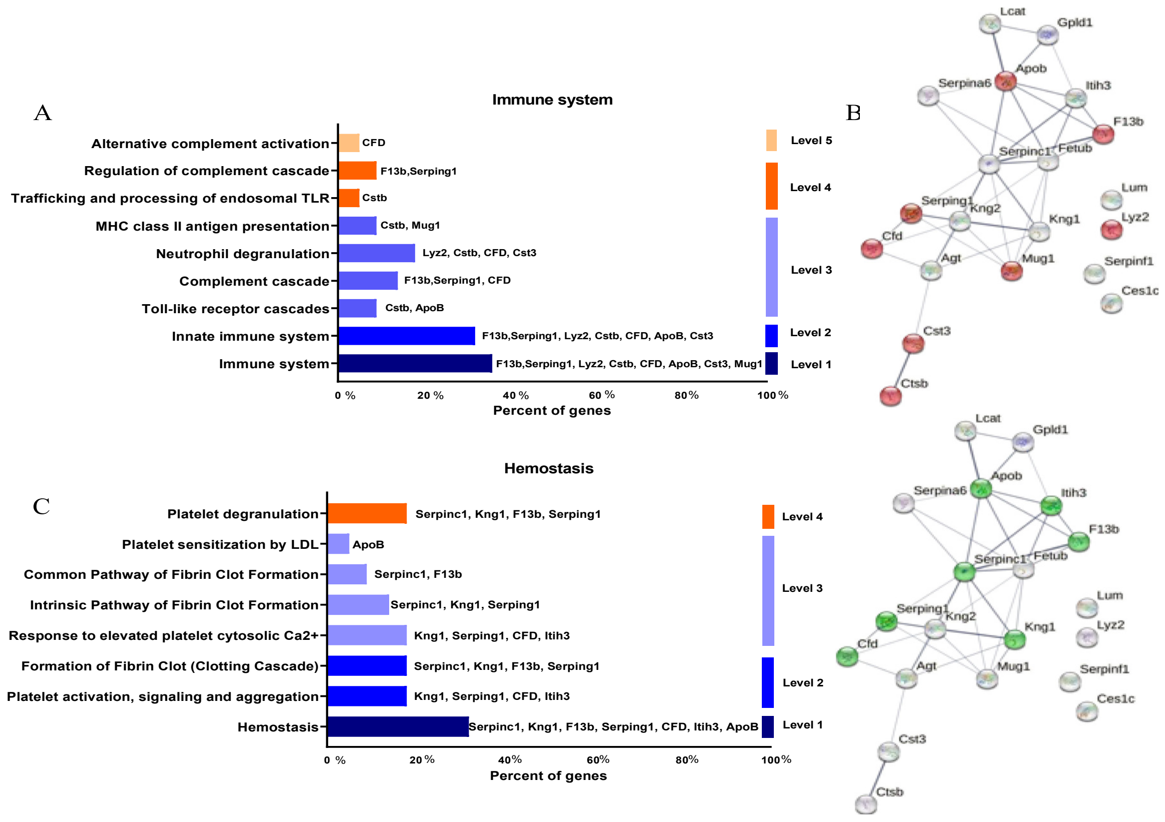

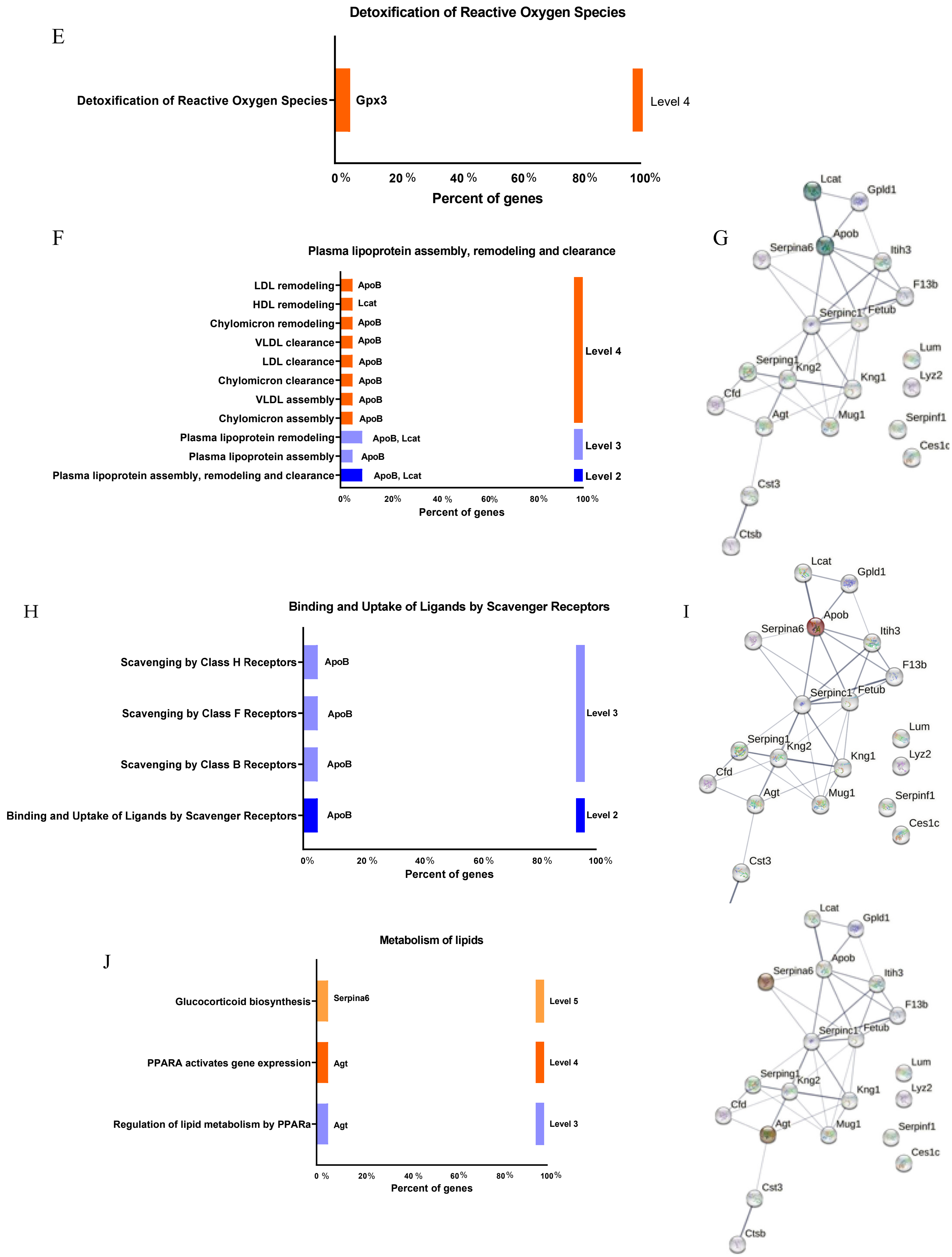
E
Detoxification of Reactive Oxygen Species
Detoxification of Reactive Oxygen Species
Gpx3
Level 4
0 %
20 %
40 %
60 %
80 %
100%
Percent of genes
F
Plasma lipoprotein assembly, remodeling and clearance
LDL remodeling
ApoB
HDL remodeling
Lcat
Chylomicron remodeling
ApoB
VLDL clearance
ApoB
LDL clearance
ApoB
Chylomicron clearance
ApoB
VLDL assembly
ApoB
Chylomicron assembly
ApoB
Plasma lipoprotein remodeling
ApoB, Lcat
Plasma lipoprotein assembly
ApoB
Plasma lipoprotein assembly, remodeling and clearance
ApoB, Lcat
Level 4
Level 3
Level 2
0%
20%
40 %
60%
80 %
100 %
Percent of genes
G
Lcat
Gpld1
Apob
Serpina6
Itih3
F13b
Serpinc1
Fetub
Lum
Serping1
Kng2
Kng1
Lyz2
Cfd
Agt
Mug1
Serpinf1
Cst3
Ctsb
H
Binding and Uptake of Ligands by Scavenger Receptors
Scavenging by Class H Receptors
ApoB
Scavenging by Class F Receptors
ApoB
Scavenging by Class B Receptors
ApoB
Binding and Uptake of Ligands by Scavenger Receptors
ApoB
Level 3
Level 2
0%
20 %
40%
60 %
80 %
100%
Percent of genes
I
Lcat
Gpld1
Apob
Serpina6
Itih3
F13b
Serpinc1
Fetub
Lum
Serping1
Kng2
Kng1
Lyz2
Cfd
Agt
Mug1
Serpinf1
Ces1c
Cst3
J
Metabolism of lipids
Glucocorticoid biosynthesis
Serpina6
Level 5
PPARA activates gene expression
Agt
Level 4
Regulation of lipid metabolism by PPARa
Agt
Level 3
0 %
20%
40 %
60 %
80 %
100 %
Percent of genes
Lcat
Gpld1
Apob
Serpina6
Itih3
F13b
Serpinc1
Fetub
Lum
Serping1
Kng2
Kng1
Lyz2
Cfd
Agt
Mug1
Serpinf1
Ces1c
Cst3
Ctsb

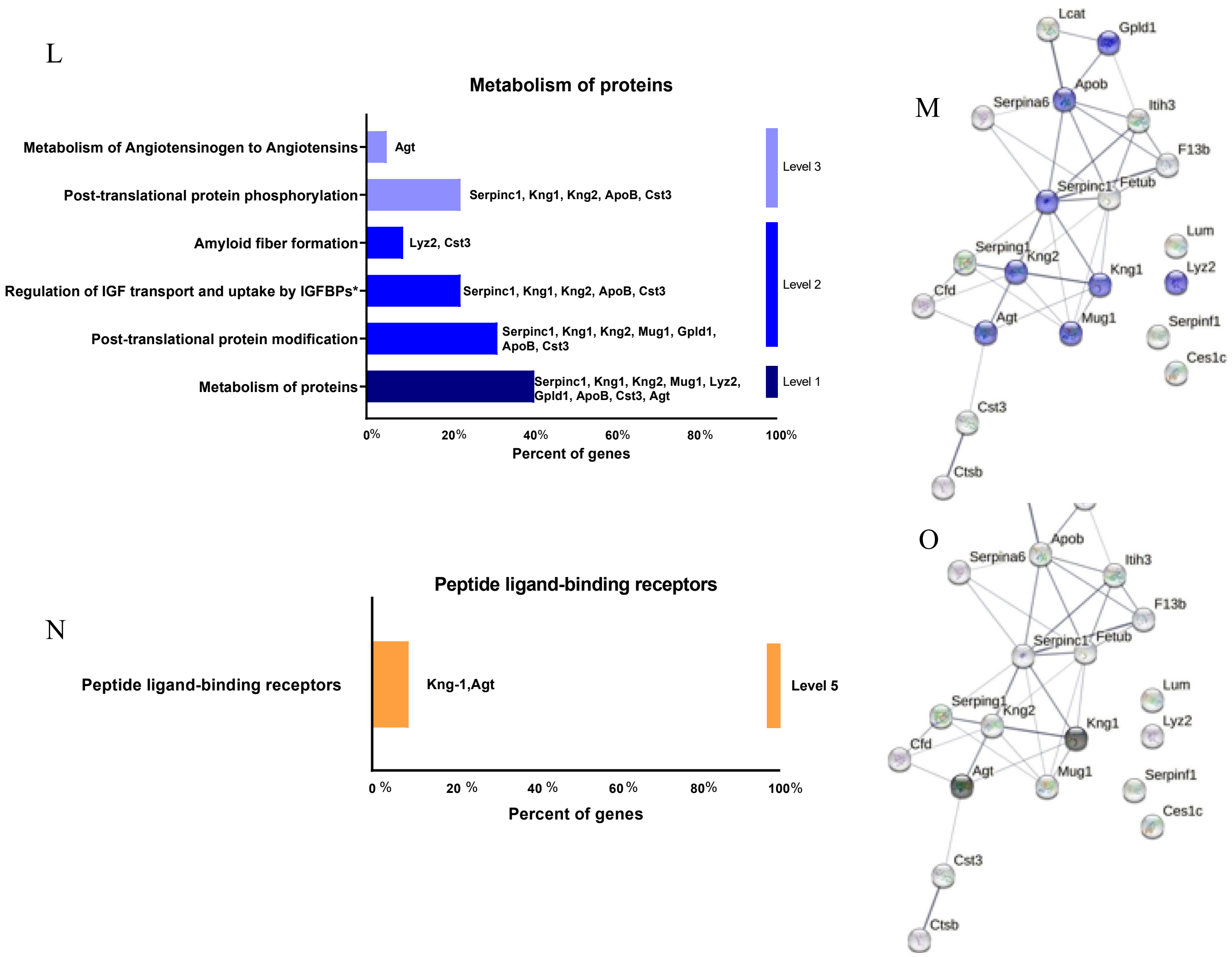
L
Metabolism of proteins
Metabolism of Angiotensinogen to Angiotensins
Agt
Post-translational protein phosphorylation
Serpinc1, Kng1, Kng2, ApoB, Cst3
Amyloid fiber formation
Lyz2, Cst3
Regulation of IGF transport and uptake by IGFBPs*
Serpinc1, Kng1, Kng2, ApoB, Cst3
Post-translational protein modification
Serpinc1, Kng1, Kng2, Mug1, Gpld1, ApoB, Cst3
Metabolism of proteins
Serpinc1, Kng1, Kng2, Mug1, Lyz2, Gpld1, ApoB, Cst3, Agt
Level 3
Level 2
Level 1
0%
20%
40%
60%
80%
100%
Percent of genes
M
Lcat
Gpld1
Apob
Serpina6
Itih3
F13b
Serpinc1
Fetub
Serping1
Kng2
Kng1
Lum
Lyz2
Cfd
Agt
Mug1
Serpinf1
Ces1c
Cst3
Ctsb
N
Peptide ligand-binding receptors
Peptide ligand-binding receptors
Kng-1,Agt
Level 5
0 %
20 %
40 %
60 %
80%
100%
Percent of genes
O
Apob
Serpina6
Itih3
F13b
Serpinc1
Fetub
Serping1
Kng2
Kng1
Lum
Lyz2
Cfd
Agt
Mug1
Serpinf1
Ces1c
Cst3
Ctsb
